# Factors affecting prostate cancer detection through asymptomatic prostate-specific antigen testing in primary care in England: evidence from the 2018 National Cancer Diagnosis Audit

**DOI:** 10.3399/BJGP.2024.0376

**Published:** 2025-02-25

**Authors:** Samuel WD Merriel, Nurunnahar Akter, Nadine Zakkak, Ruth Swann, Sean McPhail, Greg Rubin, Georgios Lyratzopoulos, Gary Abel

**Affiliations:** Centre for Primary Care and Health Services Research, University of Manchester, Manchester.; Exeter collaboration of Academic Primary Care (APEx), University of Exeter, Exeter; Department of Health Data Science, University of Liverpool, Liverpool.; University College London, London; data and research analyst, Cancer Research UK, London.; Cancer Research UK, London; NHS England, London.; National Cancer Registration and Analysis Service, NHS Digital, Leeds.; Newcastle University, Newcastle upon Tyne.; University College London, London.; APEx, University of Exeter, Exeter.

**Keywords:** asymptomatic, primary health care, prostate, prostate cancer, prostate-specific antigen

## Abstract

**Background:**

Prostate-specific antigen (PSA) is used in primary care for prostate cancer detection, either for symptomatic assessment or asymptomatic testing following an informed decision.

**Aim:**

To estimate the proportion of patients with prostate cancer who were diagnosed following asymptomatic PSA testing, and the patient and practice factors influencing this route.

**Design and setting:**

The 2018 English National Cancer Diagnosis Audit (NCDA) data were analysed, with linkage to the National Cancer Registration and Analysis Service, practice-level Quality and Outcomes Framework (QOF), and GP Patient Survey (GPPS) data. All 2018 NCDA patients with a diagnosis of prostate cancer were included (*n* = 9837).

**Method:**

Patients with recorded biomarker testing and no recorded symptoms before diagnosis were classified as having asymptomatic PSA-detected prostate cancer. Patient (age, ethnicity, deprivation, and comorbidities) and practice (geographical location, area deprivation, list size, urgent suspected cancer referral rate, QOF outcomes, and GPPS results) factors were analysed for association with asymptomatic PSA testing using mixed-effects logistic regression models.

**Results:**

In total, 1884 out of 9837 (19.2%) patients with prostate cancer were detected following asymptomatic PSA testing, 982 (52.1%) of whom were aged 50–69 years. Younger age, non-White ethnicity, lower deprivation, and lower comorbidity count were associated with an increased likelihood of diagnosis following asymptomatic PSA testing. There was a 13-fold variation between practices in the odds of detecting prostate cancer through asymptomatic PSA testing, without clear explanatory practice-level factors.

**Conclusion:**

One in five patients with prostate cancer in England are diagnosed after asymptomatic PSA testing in primary care, with large variation in asymptomatic PSA detection between practices.

## Introduction

Prostate cancer is the most commonly diagnosed type of cancer for men in the UK, with over 50 000 new patients diagnosed with prostate cancer each year. Early-stage diagnosis of prostate cancer remains a challenge, with just over 51% of patients diagnosed at stage 1 or 2 in 2020 in England. Referrals from primary care remain the most common route to diagnosis for prostate cancer. In 2018, 57.2% of patients were diagnosed from an urgent suspected cancer (formerly 2-week wait) referral, with a further 24.8% diagnosed after a routine GP referral. Emergency diagnosis of prostate cancer remains relatively rare (6.1% of all new patient cases).^1^

Prostate-specific antigen (PSA) testing is the only test available in UK primary care for prostate cancer detection. PSA is used as part of symptomatic assessment for men presenting with lower urinary tract symptoms (LUTS), erectile dysfunction, or haematuria.^2^ The UK National Screening Committee currently recommends against a national PSA-based prostate cancer screening programme.^3^ Instead, the UK Government Office for Health Improvement and Disparities introduced the prostate cancer risk management programme for asymptomatic men aged ≥50 years who request PSA testing in primary care after making an informed decision about the potential benefits and harms of testing.^4^ Critics of asymptomatic PSA testing suggest it has no benefits in terms of mortality reduction but is still associated with overdiagnosis and worsening health inequalities.^5^ Uncertainty remains regarding the current levels of asymptomatic PSA testing from studies based on coded clinical data;^6^^,^^7^ however, regional variation in PSA thresholds is estimated to have produced significant disparities in prostate cancer referrals, diagnostic testing, and detection rates across England.^8^

**Table:** How this fits in

Asymptomatic, informed-choice prostate-specific antigen (PSA) testing occurs in primary care in the UK in the absence of a national prostate cancer screening programme. This study shows that four-fifths of prostate cancers are diagnosed following symptomatic presentation rather than from asymptomatic PSA testing in England. There is a 13-fold variation in asymptomatic PSA test-detected prostate cancer between English GP practices, without clear explanatory practice-level factors. Patient factors among men diagnosed with prostate cancer, including ethnicity, age, deprivation, and multimorbidity, have a significant impact on the likelihood of being diagnosed following asymptomatic PSA testing.

The National Cancer Diagnosis Audit (NCDA) aimed to assess the diagnostic process for cancer among patients presenting to primary care, identify best practice, and inform quality improvement activities. Details of the data collection, which involved clinicians auditing coded and free-text electronic healthcare records data, has been outlined in previous publications.^9^^,^^10^ The current study aimed to analyse the 2018 NCDA data to understand factors affecting asymptomatic PSA testing in English primary care before diagnosis for patients with prostate cancer.

## Method

The 2018 NCDA dataset was used to investigate the use of primary care cancer biomarker tests based on asymptomatic status in individuals who were subsequently diagnosed with prostate cancer. The information on the use of diagnostic tests and other characteristics of patients diagnosed with cancer in 2018 was collected between 2019 and 2020 by participating general practices using primary care records and the National Cancer Registration and Analysis Service. The patients in the audit were representative of the national incident cohort of patients with cancer in terms of age, gender, stage of cancer, and cancer site, and the characteristics of the participating practices were comparable with non-participating practices.^9^ Of the 33 359 men included in the 2018 NCDA, 9837 were diagnosed with prostate cancer and were selected for this study.

For the purposes of this study, patients were defined as having an ‘asymptomatic PSA-detected diagnosis’ on the basis of meeting two conditions. First, they must have no evidence of presenting symptoms recorded in the audit data and thus the patient is assumed to be asymptomatic (entries of not known or not applicable were treated as an absence of symptoms). Second, patients must have had a primary care-led cancer biomarker test recorded in the audit. The audit does not specify the type of biomarker used, but in men it was assumed that this related to PSA testing. A binary variable was created with patients meeting both conditions coded as asymptomatic PSA-detected, and patients meeting only one or neither condition coded as symptomatic. Data were also available on presenting signs or abnormal test results, but these were not used in the definition as it could not be determined if they were observed before or after PSA testing. A wide range of exposure variables were considered, including various patient characteristics and practice factors.

Two mixed-effect logistic regression models were fitted to examine variation in asymptomatic prostate cancer detection. The first model (model A) adjusted for the following patients’ characteristics: ethnicity (White or non-White), age group (35–49, 50–59, 60–69, 70–79, and ≥80 years), deprivation quintile (based on the 2019 Index of Multiple Deprivation of lower layer super output area of English residence), and count of comorbidities (0, 1, 2, 3, and ≥4), including general practice as a random effect.

The second model (model B) incorporates the variables included in model A along with the following practice factors: practice-level deprivation quintile; rurality (urban or rural); practice list size (size 1: <6000 patients, size 2: 6000–12 000, and size 3: >12 000);^11^ urgent suspected cancer (USC) referral rate for cancer diagnosis;^12^ patients per practice; Quality and Outcomes Framework score;^13^ percentage of registered patients aged ≥65 years; and variables representing key aspects of patients’ experience (access, continuity, satisfaction, and doctor communication), as reported in the 2018 GP Patient Survey (GPPS).^14^

To ensure that the findings were not driven by emergency or late-stage diagnoses without providing an opportunity for asymptomatic detection, a sensitivity analysis was performed including all patient characteristics adjusted in model A in addition to the stage of cancer (1, 2, 3, and 4), and whether patients were diagnosed as an emergency or not (derived from the patient’s final route of diagnosis). To facilitate comparisons of the effect sizes, continuous variables were standardised before fitting the model so that the odds ratios (ORs) estimated from the regression models correspond to one standard deviation change in the exposure variables. All statistical analyses were conducted in Stata SE (version 17).

## Results

**Figure 1.**
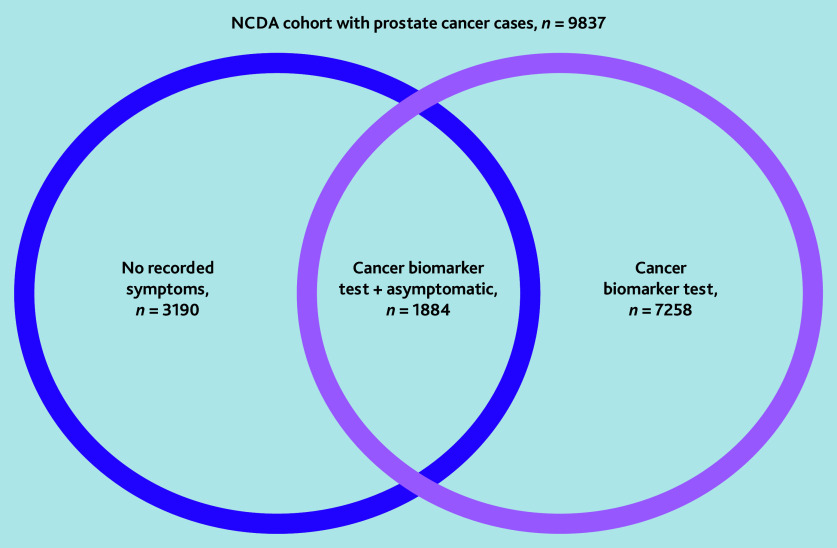
Patient selection from English NCDA cohort with prostate cancer. NCDA = National Cancer Diagnosis Audit.

The analysis sample consisted of 9837 men. diagnosed with prostate cancer from 1639 general practices. Of 9837 patients with prostate cancer, 73.8% (*n* = 7258/9837) underwent a cancer biomarker test; 32.4% (*n* = 3190/9837) were asymptomatic at diagnosis (of which 837 were recorded as symptoms being ‘not known’ and 2353 were ‘not applicable’); and 19.2% (*n* = 1884/9837) had experienced both (Figure 1). In total, 73.1% (*n* = 1377/1884) of patients in the asymptomatic PSA-detected group had an abnormal PSA result recorded in the audit presenting signs or abnormal test field, and only 8.3% (*n* = 156/1884) had a sign or test result other than raised PSA, ‘other’, ‘not applicable’, or ‘not known’. In 3.2% (*n* = 60/1884) of patients a prostate mass was recorded.

Table 1 shows the characteristics of patients with prostate cancer based on whether they had a recorded symptom status. The proportion of patients who were asymptomatic was greater in non-White patients than White patients (22.5% versus 18.5%). The most deprived population group had the lowest proportion of patients diagnosed following asymptomatic PSA testing compared with the least deprived (21.3% versus 15.4%). The highest proportion of patients diagnosed following asymptomatic PSA testing was in the youngest age group and the lowest was in the oldest age group (35–49 years, 29.8%; ≥80 years, 12.7%). Patients with no chronic condition or fewer comorbidities (≤2) as well as patients in early stages of cancer (stage 1 and 2) were more likely to be diagnosed following asymptomatic PSA testing than those with more comorbidites or diagnosed at late stage.

**Table 1.** Characteristics of patients with prostate cancer based on symptom status

**Characteristic**	**Asymptomatic PSA-detected patients, *n* = 1884, *n* (row %)**	**Symptomatic patients, *n* = 7953, *n* (row %)**	**Total, *N* = 9837, *n***	***P*-value^a^**
**Ethnicity**				0.003
White	1546 (18.5)	6794 (81.5)	8340	
Non-White	208 (22.5)	716 (77.5)	924	
Missing	130 (22.7)	443 (77.3)	573	

**Age group, years**				<0.001
35–49	31 (29.8)	73 (70.2)	104	
50–59	294 (23.6)	950 (76.4)	1244	
60–69	688 (21.2)	2555 (78.8)	3243	
70–79	673 (18.2)	3018 (81.8)	3691	
≥80	198 (12.7)	1357 (87.3)	1555	

**IMD quintile**				<0.001
1, least deprived	508 (21.3)	1882 (78.7)	2390	
2	418 (19.0)	1777 (81.0)	2195	
3	387 (19.9)	1555 (80.1)	1942	
4	333 (18.8)	1434 (81.2)	1767	
5, most deprived	238 (15.4)	1305 (84.6)	1543	

**Comorbidities**				<0.001
0	481 (23.7)	1552 (76.3)	2033	
1	668 (20.5)	2595 (79.5)	3263	
2	436 (17.9)	2006 (82.1)	2442	
3	178 (14.6)	1044 (85.4)	1222	
≥4	121 (13.8)	756 (86.2)	877	

**Stage of cancer**				<0.001
1	786 (22.0)	2780 (78.0)	3566	
2	331 (24.9)	999 (75.1)	1330	
3	452 (19.7)	1847 (80.3)	2299	
4	159 (9.0)	1604 (91.0)	1763	
Missing	156 (17.7)	723 (82.3)	879	

a P-*values are from a Pearson χ^2^-test of association. IMD = Index of Multiple Deprivation.*

*PSA = prostate-specific antigen.*

Table 2 shows the results from adjusted models estimating the odds of being asymptomatic and having a primary care biomarker test among patients with prostate cancer. In model A, there was weak evidence (*P* = 0.046) that non-White patients were more likely to be in the asymptomatic PSA-detected group than White patients (OR 1.21, 95% confidence interval [CI] = 1.01 to 1.46). Older age was associated with having a lower chance of being in the asymptomatic PSA-detected group, with ORs of 0.88 (95% CI = 0.77 to 1.01) and 0.59 (95% CI = 0.49 to 0.72) in the 70–79 years and ≥80 years age groups, respectively, compared with the 60–69 years age group.

**Table 2.** Adjusted ORs and 95% CIs estimating the likelihood of asymptomatic PSA-detected prostate cancer in primary care patients

**Exposure and variables**	**Model A^a^**		**Model B^b^**	

	**OR (95% CI)**	***P*-value**	**OR (95% CI)**	***P*-value**
**Ethnicity**				
White	Reference		Reference	
Non-White	1.21 (1.00 to 1.46)	0.046	1.20 (0.99 to 1.45)	0.067

**Age group, years**				
35–49	1.31 (0.79 to 2.16)		1.22 (0.71 to 2.10)	
50–59	1.11 (0.93 to 1.33)		1.11 (0.93 to 1.34)	
60–69	Reference	<0.001	Reference	<0.001
70–79	0.88 (0.77 to 1.01)		0.88 (0.77 to 1.01)	
≥80	0.59 (0.49 to 0.72)		0.60 (0.50 to 0.74)	

**IMD quintile**				
1, least deprived	Reference		Reference	
2	0.89 (0.76 to 1.05)		0.90 (0.76 to 1.07)	
3	0.91 (0.76 to 1.08)	0.01	0.89 (0.75 to 1.06)	0.015
4	0.84 (0.70 to 1.01)		0.86 (0.72 to 1.03)	
5, most deprived	0.69 (0.57 to 0.85)		0.69 (0.56 to 0.85)	

**Comorbidities**				
0	Reference		Reference	
1	0.85 (0.73 to 0.98)		0.83 (0.71 to 0.97)	
2	0.76 (0.64 to 0.89)	<0.001	0.76 (0.63 to 0.91)	<0.001
3	0.63 (0.51 to 0.78)		0.65 (0.52 to 0.81)	
≥4	0.59 (0.46 to 0.75)		0.60 (0.47 to 0.76)	

**Rurality**				
Urban			Reference	
Rural			0.93 (0.72 to 1.20)	0.562

**Practice list size**				
Size 1, <6000			Reference	
Size 2, 6000–12 000			1.02 (0.85 to 1.22)	0.073
Size 3, >12 000			1.24 (1.00 to 1.54)	

**GPPS access**			1.00 (0.87 to 1.45)	0.988

**GPPS continuity**			1.12 (1.05 to 1.26)	0.044

**GPPS satisfaction**			0.89 (0.71 to 1.09)	0.252

**GPPS communication**			1.05 (0.90 to 1.21)	0.535

**USC referral**			1.01 (0.93 to 1.08)	0.886

**Maximum QOF**			1.04 (0.97 to 1.21)	0.277

**Patients per GP**			0.97 (0.89 to 1.04)	0.402

**Patients aged >65 years per GP**			0.95 (0.87 to 1.03)	0.234

a *Adjusted for patient factors (ethnicity, age, deprivation, and number of morbidities).*

b *Adjusted for patient factors (as per model A) and practice factors (rurality, list size, GPPS scores, USC referral rate, QOF points, patients per full-time equivalent GP, and the proportion of patients aged >65 years). GPPS = GP Patient Survey. OR = odds ratio. PSA = prostate-specific antigen. QOF = Quality and Outcomes Framework. USC = urgent suspected cancer.*

There was also a lower tendency for asymptomatic PSA-detected prostate cancer among patients with a higher level of deprivation (OR for most versus least deprived: 0.69, 95% CI = 0.57 to 0.85, *P* = 0.01). Patients with an increasing number of chronic medical conditions had lower odds of asymptomatic PSA-based detection compared with patients with no medical condition (OR for ≥4 comorbidities versus 0 comorbidities: 0.59, 95% CI = 0.46 to 0.75, *P*<0.001) (Table 2).

In model A, the estimated standard deviation of between-practice variation was 0.66 (95% CI = 0.57 to 0.77), which translates to a 95% mid-range OR of 13.4, that is, an over 13-fold variation in the odds of detecting prostate cancer through asymptomatic PSA testing between practices with the highest and lowest rates (ignoring the 5% of extreme practices). In model B, this practice-level standard deviation was 0.63 (95% CI = 0.53 to 0.74), indicating that little of the between-practice variation could be explained by the practice factors in the model. Indeed, the only practice factor that was statistically significant was continuity of care (as measured by the GPPS), which indicated that practices with higher scores for continuity of care had higher rates of patients in the asymptomatic PSA-detected case group.

In the sensitivity analysis (see Supplementary Table S1) after additionally adjusting for stage of cancer and final route of diagnosis in model A, the ORs for patient characteristics attenuated somewhat and ethnicity and deprivation were no longer significant. Patients with stage 4 cancer had lower odds of asymptomatic PSA detection (OR 0.40, 95% CI = 0.33 to 0.49, *P*<0.001) compared with patients with stage 1 cancer. Similarly, patients whose final route of diagnosis was emergency had a lower chance (OR 0.30, 95% CI = 0.19 to 0.46, *P*<0.001) than the patients whose final route was non-emergency.

## Discussion

### Summary

This study of 9837 men with prostate cancer found that nearly one in five (19.2%) were diagnosed after asymptomatic PSA testing. The study found that 46.2% (*n* = 871/1884) of patients who were asymptomatic and diagnosed with prostate cancer were aged ≥70 years at the time of diagnosis. Patient factors strongly affected the route to prostate cancer detection (asymptomatic PSA testing versus symptomatic detection). Younger men, patients with fewer comorbidities, and people from less deprived areas were more likely to be detected from asymptomatic PSA testing. Men from non-White backgrounds were more likely to be diagnosed after asymptomatic PSA testing compared with White men.

However, the strongest predictor of whether a man was asymptomatically detected following a PSA test was the practice he was registered at (identified from the random intercept). Significant variation exists in the likelihood of asymptomatic PSA-detected prostate cancer between GP practices. The study did not identify any particular practice factors that influenced the likelihood of asymptomatic prostate cancer detection, apart from higher levels of continuity, which were weakly associated with more prostate cancers diagnosed asymptomatically.

### Strengths and limitations

There are a number of strengths to this study. A rich depth of data was secured through the NCDA methodology of clinicians reviewing the entire primary care electronic health record, including free-text data, to understand the patient’s cancer diagnostic journey. Linkage to the national cancer registry and patient surveys enriches the findings and was used to explore a wider range of factors affecting the routes to diagnosis for prostate cancer. The patient population within the NCDA is also broadly representative of patients diagnosed with cancer within the UK.^9^

There are also some limitations to take into consideration. The ideal study would have measured whether PSA was used as an asymptomatic test or in response to symptoms directly, nonetheless this study used an operational definition to identify such use. The NCDA only captured data from patients with cancer, so this study is unable to estimate the levels of PSA testing being done in primary care and the proportion of patients undergoing testing who are diagnosed with prostate cancer. Symptom data may not always be captured in a patient’s record if they opt to undertake PSA testing during a routine phlebotomy appointment for other reasons, affecting the accuracy of estimates around symptomatic versus asymptomatic testing.

Furthermore, in this study the authors assumed that a PSA test in the absence of recorded symptoms always led to a diagnosis. However, this may not be true in a small number of patients’ cases — for example, 38 such patients were diagnosed as an emergency, which is unlikely to occur as a direct result of a PSA test. Test data were only reported as test type (biomarker, blood, urinary, imaging, stool, or endoscopy), so it is unclear whether biomarker tests before prostate cancer diagnosis were all PSA tests. However, PSA is the only test currently available for prostate cancer detection in primary care and drives eligibility for USC referral for diagnostic testing. Therefore, it is very likely that almost all biomarker tests before prostate cancer diagnosis included PSA. This is further supported by the high rate of abnormal PSA results recorded for these patients. Although a small number of these patients had other signs or abnormal test results recorded, it is likely that many were observed either concurrent with (for blood tests) or following (for prostate mass) abnormal PSA findings.

### Comparison with existing literature

Two previous studies have been conducted using routinely collected primary healthcare records to estimate the proportions of patients in primary care undergoing asymptomatic PSA testing in the UK. Young *et al* analysed data from the Clinical Practice Research Datalink GOLD on 450 000 men from all regions of the UK outside of London and inferred from the patterns of PSA testing that the majority was for symptomatic assessment.^7^ Clift *et al* concluded from an analysis of the QResearch database that 65.1% of PSA testing in UK primary care between 1997 and 2017 was for asymptomatic patients.^6^ Both studies relied on coded data, which may be incomplete.^15^ The NCDA captures both coded and free-text data through review of the patient’s medical records by a clinician, which increases the likelihood that a more accurate picture can be established from the current study.

A number of patient factors were found to affect the likelihood of asymptomatic PSA testing in UK primary care. PSA testing is known to be less common in more deprived populations,^16^^–^19 as was the case in this study. Data from Australia, Canada, and some Western Europe countries suggest PSA screening is increasingly common with older age.^16^^,^^20^^–^22 NCDA data suggest asymptomatic prostate cancer diagnosis following PSA testing is less common compared with symptomatic detection in England as age increases, which may reflect the increasing incidence of LUTS with age rather than PSA test rates. Nderitu *et al* analysed data from GP practices in inner East London and showed that Black and South Asian men were more likely to undergo PSA testing compared with White men,^17^ which is consistent with the current study. The association with GP continuity and PSA screening identified in the NCDA is also consistent with findings from the Canadian Community Health Survey between 2009 and 2014.^16^

### Implications for practice

This study highlights some key areas for potential improvement for prostate cancer detection in primary care. The 13-fold variation in rates of asymptomatic PSA-detected prostate cancer between practices speaks to the ongoing lack of clarity regarding prostate cancer screening practice in the UK.^23^ Evidence from this study and others consistently show that men from more deprived areas are less likely to undergo PSA testing and more likely to be diagnosed with late-stage prostate cancer.^6^^,^^24^ Patient factors were found to have a profound impact on PSA testing and are, therefore, likely to play a role in driving prostate cancer inequalities. A more consistent approach to asymptomatic PSA testing with initiatives to raise awareness about prostate cancer, communicating the pros and cons of undergoing PSA testing among men, and addressing barriers to accessing primary care may help to reduce such inequalities.

The benefits of an informed choice model for asymptomatic PSA testing compared with organised prostate cancer screening programmes are questionable at best.^5^ Current UK guidance for asymptomatic PSA testing lacks any nuance with regards to testing men at higher risk, nor does it recognise there is a point in terms of age at which the harms of asymptomatic PSA testing outweigh the benefits.^4^ In this study, almost half of those patients where prostate cancer was detected through asymptomatic PSA testing were in men aged ≥70 years. Although it must be recognised that prostate cancer incidence in the UK peaks in men in their late 70s and some men in their 70s are relatively fit and well, better information is needed for men and GPs to decide when asymptomatic PSA testing may cause more harm than good.

In conclusion, NCDA data show that asymptomatic PSA testing accounts for the minority of patients diagnosed with prostate cancer through primary care in England. Significant variation in detection of individuals with asymptomatic prostate cancer persists between GP practices. Patient factors, including age, ethnicity, deprivation, and comorbidities, appear to affect the likelihood of prostate cancer diagnosis following asymptomatic PSA testing.

## Data

The NCDA dataset is available through NHS England’s Data Access Request Service to those able to demonstrate that they can meet all of NHS England’s legal, ethical, and security requirements for safe data research.
